# Evidence for reduced neurogenesis in the aging human hippocampus despite stable stem cell markers

**DOI:** 10.1111/acel.12641

**Published:** 2017-08-01

**Authors:** Kathryn J. Mathews, Katherine M. Allen, Danny Boerrigter, Helen Ball, Cynthia Shannon Weickert, Kay L. Double

**Affiliations:** ^1^ Discipline of Biomedical Science and Brain and Mind Centre Sydney Medical School The University of Sydney Sydney NSW 2006 Australia; ^2^ Neuroscience Research Australia Randwick NSW 2031 Australia; ^3^ Schizophrenia Research Institute Randwick NSW 2031 Australia; ^4^ Biostatistics and Bioinformatics Facility Bosch Institute The University of Sydney Sydney NSW 2006 Australia; ^5^ School of Psychiatry The University of New South Wales Sydney NSW 2052 Australia

**Keywords:** cognition, doublecortin, healthy aging, hippocampus, Ki67, neurogenesis

## Abstract

Reduced neurogenesis in the aging mammalian hippocampus has been linked to cognitive deficits and increased risk of dementia. We utilized postmortem human hippocampal tissue from 26 subjects aged 18–88 years to investigate changes in expression of six genes representing different stages of neurogenesis across the healthy adult lifespan. Progressive and significant decreases in mRNA levels of the proliferation marker Ki67 (*MKI67*) and the immature neuronal marker doublecortin (*DCX*) were found in the healthy human hippocampus over the lifespan. In contrast, expression of genes for the stem cell marker glial fibrillary acidic protein delta and the neuronal progenitor marker eomesodermin was unchanged with age. These data are consistent with a persistence of the hippocampal stem cell population with age. Age‐associated expression of the proliferation and immature neuron markers *MKI67* and *DCX,* respectively, was unrelated, suggesting that neurogenesis‐associated processes are independently altered at these points in the development from stem cell to neuron. These data are the first to demonstrate normal age‐related decreases at specific stages of adult human hippocampal neurogenesis.

## Introduction

Neurogenesis persists in the subgranular zone of the adult human hippocampus and arises from a pool of quiescent stem cells which, if appropriately stimulated, undergo proliferation and subsequent maturation into neurons. These new neurons are thought to play an important role in normal hippocampal function, particularly in the ongoing maintenance of hippocampus‐dependent spatial and declarative memory (Aimone *et al*., 2014; Christian *et al*., 2014). It has therefore been hypothesized that alterations in hippocampal neurogenesis may be a contributing factor to cognitive decline and dementia. This is supported by observations that radiation‐induced ablation of hippocampal neurogenesis in rats results in cognitive decline (Monje *et al*., 2002) and clinical evidence of cognitive changes in patients receiving radiation therapy and chemotherapy (Monje & Dietrich, 2012). Further evidence of a relationship between hippocampal neurogenesis and cognitive function is provided by disorders associated with cognitive decline, including Alzheimer's disease (Boekhoorn *et al*., 2006) and Parkinson's disease (Hoglinger *et al*., 2004) where stem cell proliferation and/or nascent neuron maturation is altered.

**Figure 1:**
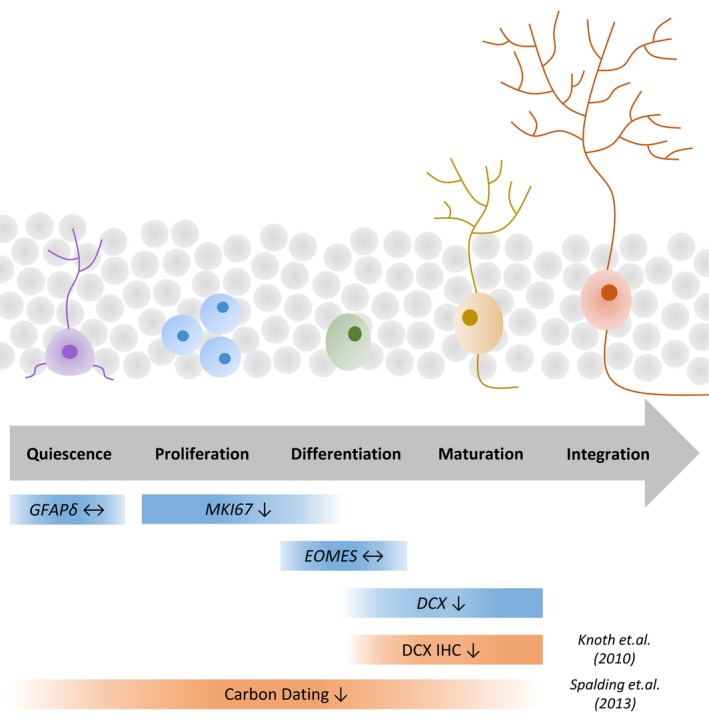
Staging neurogenesis using transcripts representative of different developmental stages from stem cell to neuron. Neurogenesis‐associated genes are variably expressed throughout development from stem cell to functional neuron. In this study, expression of genes (blue) was chosen for their ability to identify discrete points of neurogenesis with minimal cross‐over between developmental stages. Previous studies of hippocampal neurogenesis over the human lifespan (orange) have reported either late‐stage development of neurons only or the broad birth of neurons without identifying different stages of neurogenesis. Arrows represent age‐related reductions (↓) or unchanged (↔) expression of the markers. *GFAP*δ – glial fibrillary acidic protein delta; *MKI67*, Ki67; *EOMES,* eomesodermin; *DCX,* doublecortin; IHC, immunohistochemistry.

The birth of new neurons is a complex, multistep process, any stage of which could potentially be altered with age (Fig. 1). Reduced neurogenesis in the aged rodent hippocampus has been attributed to impaired proliferation and maturation of neuronal precursors (Rao *et al*., 2006). The two available studies of neurogenesis in the aging human hippocampus using immunohistochemical (Knoth *et al*., 2010) and carbon dating (Spalding *et al*., 2013) techniques suggest that hippocampal neurogenesis is progressively reduced with age but employed markers that were either relatively restricted to a specific developmental stage or that broadly identified new cell birth (Fig. 1). To date, no studies have investigated the progression of neurogenesis through quiescence, proliferation, differentiation and maturation in the same hippocampal tissue over the healthy adult lifespan. Appreciating at which stage neurogenesis may fail with age in the human brain is critical to the development of strategies that aim to preserve neurogenesis and thus, cognitive function. Studies in aged rodents consistently report that interventions including exercise (Van Praag *et al*., 2005), environmental enrichment (Kempermann *et al*., 2002), inflammatory blockade (Monje *et al*., 2003; Ormerod *et al*., 2013; Speisman *et al*., 2013) or hormonal modulation (Montaron *et al*., 1999) are associated with both increased neurogenesis and improved cognition. The potential of similar interventions, such as exercise or cognitive training, in maintaining cognitive function in the healthy aged human brain is now recognized (Ballesteros *et al*., 2015)**,** although for technical reasons linking these findings to human neurogenesis is more difficult. Such approaches will, however, be most effective if an adequate pool of quiescent stem cells are available to be influenced for therapeutic benefit.

Despite these promising avenues of research, the effect of age upon the hippocampal stem cell population in the rodent brain remains a matter of debate and has not been explored in the human hippocampus. Hippocampal stem cells in the rodent brain were reported to possess no means of self‐renewal (Encinas *et al*., 2011), suggesting that a stem cell deficit may underlie the observed progressive decline in neurogenesis with age (Walter *et al*., 2011; Andersen *et al*., 2014). In contrast, other studies reported that hippocampal stem cells in the rodent brain are capable of self‐renewal (Suh *et al*., 2007) and that this ability persists throughout adulthood (Montaron *et al*., 1999; Hattiangady & Shetty, 2008; Bonaguidi *et al*., 2011), which may thus represent a tractable target for restoring age‐related decreases in neurogenesis. In this study, we aimed to investigate the effect of age upon multiple stages of neurogenesis by analysing changes to neurogenesis‐associated gene expression across the healthy adult human lifespan.

Gene transcripts were chosen to estimate the proportion of cells at different stages of neurogenesis (Fig. 1, Table S2). Markers for stem cells (glial fibrillary acidic protein isoform delta; *GFAP*δ), cell proliferation (Ki67; *MKI67*), neuronal progenitor cells (eomesodermin; *EOMES*), immature neurons (doublecortin; *DCX*) and mature astrocytes (S100 calcium‐binding protein B; *S100B* and glial fibrillary acidic protein; *GFAP –* although GFAP also marks stem cells) were quantified using quantitative reverse transcriptase–polymerase chain reaction (qRT–PCR) from total RNA extracted from the hippocampus of 26 normal individuals aged 18–88 (Table S1, Data S1).

**Figure 2:**
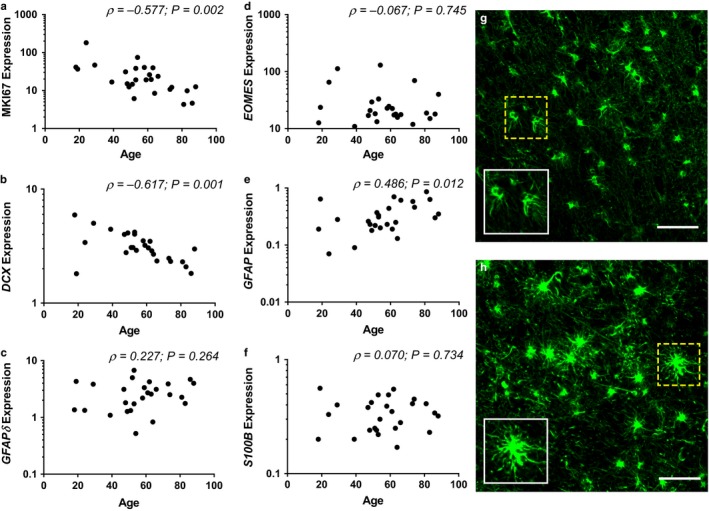
Alterations in neurogenesis gene expression and astrocytic activation state with age. Expression of marker Ki67 (*MKI67*) (a) and doublecortin (*DCX*) (b) was significantly reduced with age; in contrast, expression of glial fibrillary acidic protein isoform delta (*GFAP*δ) (c) and eomesodermin (*EOMES*) (d) did not vary with age. *GFAP* expression (e) significantly increased with age, but expression of *S100B* was unchanged (f), suggesting that the rate of astrogliogenesis was unvaried. Astrocyte morphology was examined using immunofluorescence in a subset of five cases as indicated in Table S1 (supporting information). Astrocytes within the hippocampus of younger individuals (g; representative image from an 18‐year‐old) exhibited typical astrocyte morphology with narrow, radiating processes and small cell bodies. Astrocytes within the hippocampus of older individuals (h; representative image from a 73‐year‐old) primarily presented with larger cell bodies strongly stained for GFAP and thick radiating processes, consistent with the morphology of activated astrocytes. Scale bar = 75 μm. Inset: magnified images of the field bound by the dotted box.

In this study, we found that expression of genetic markers for cellular proliferation (*MKI67;* ρ(26) = −0.577; *P* = 0.002, Fig. 2a) and neuronal maturation (*DCX;* ρ(26) = −0.617; *P* = 0.001, Fig. 2b) declined significantly with age. Immunohistochemistry studies in both humans and rodents report declining protein expression of these markers with age (Rao *et al*., 2006; Knoth *et al*., 2010), and we show here that these findings likely reflect transcriptional changes preceding protein synthesis. We found no significant relationship between our markers of proliferation and of immature neurons (*MKI67* and *DCX* mRNAs) when controlling for age (partial correlation, r(23) = −0.162; *P* = 0.439), suggesting that the decline in expression of these genes represents independent changes. In contrast to *MKI67* and *DCX* mRNAs, expression of the stem cell marker *GFAP*δ (ρ(26) = 0.227; *P* = 0.264, Fig. 2c) and the neuronal progenitor marker *EOMES* (ρ(26) = −0.067; *P* = 0.745, Fig. 2d) was unchanged with age. Expression of *GFAP* was significantly increased with age (ρ(26) = 0.486; *P* = 0.012; Fig. 2e), while *S100B* was unchanged with age (ρ(26) = 0.070; *P* = 0.734; Fig. 2f).

In order to explore the potential persistence of the hippocampal stem cell population with age, we investigated the expression of *GFAP*δ*,* an isoform of *GFAP* associated with quiescent stem cells (Roelofs *et al*., 2005). *GFAP*δ mRNA upregulation concurrent with *GFAP* mRNA upregulation has been reported (Roelofs *et al*., 2005) and has also been noted at the protein level in reactive astrocytes (Roelofs *et al*., 2005; Martinian *et al*., 2009). A partial correlation of age with *GFAP*δ expression, controlling for *GFAP* expression, however, found no relationship between these factors, (*r*(23) = −0.001; *P* = 0.995), demonstrating that expression of *GFAP*δ is independent of age despite the age‐associated increase in *GFAP*. Our finding of consistent *GFAP*δ expression, coupled with significantly reduced proliferation, suggests therefore that the stem cell pool in the aging human hippocampus is not depleted over time. Rather, intra‐ or extracellular factors triggering or supporting the conversion of stem cells to rapidly dividing precursors may be progressively altered with age. While further studies quantifying stem cell numbers in the aging human hippocampus will be invaluable to confirm this hypothesis, here we report data suggesting that declining neurogenesis with age may not be the result of stem cell depletion in the human hippocampus. Rather, our data are consistent with specific mechanistic changes at two independent stages of neurogenesis which influence proliferation and neuronal maturation.

We also found that expression of *DCX* decreases significantly with age, consistent with previous studies of decreased *DCX* expression in the aged human subventricular zone (Weissleder *et al*., 2016). In our study in the hippocampus, the decline in *DCX* mRNA was accompanied by consistent expression of *EOMES* (*Tbr2* in rodents), a transcription factor involved in neuronal fate decisions and expressed transiently in differentiating neurons in rodent studies (Hodge *et al*., 2012). These data suggest that neuronal progenitor cells, like quiescent stem cells, maintain a consistent population in which early neuronal specification is intact but where overall progenitor proliferation may be reduced. Reduced *DCX* expression, in conjunction with stable *EOMES* expression, suggests an unknown alteration in the hippocampal microenvironment specifically affecting the expression of genes influencing the successful maturation and migration of new neurons into the existing hippocampal circuitry. Alternatively, it may also indicate an uncontrolled exit from the immature neuronal state via early terminal differentiation or excessive cell death.

Our data suggest that the early and intermediate phases of neurogenesis (represented by *GFAP*δ and *EOMES*) are unchanged, while the proliferation and early maturation stages are significantly reduced. We speculate that neurogenesis is altered in at least two distinct stages with age. The lack of any direct correlation between reduced cell proliferation and immature neuron markers supports the idea that the changes in neurogenesis at those stages may be the result of two separate physiological changes in the aging human brain.

Neuroinflammation is one such factor which may differentially alter distinct stages of neurogenesis, being increasingly chronic in the aging hippocampus (Barrientos *et al*., 2015) and previously shown to modulate neurogenesis in *in vivo* rodent models (Ekdahl *et al*., 2003; Monje *et al*., 2003). While neuroinflammation is driven primarily by activated microglia, activated astrocytes have also recently been implicated in the regulation of the neuroinflammatory response (Jang *et al*., 2013). Experimentally, astrocyte activation is evidenced by increased *GFAP* mRNA (Nichols *et al*., 1993) and protein (David *et al*., 1997) in humans as well as morphological changes including hypertrophy and thickened radial processes (Rodríguez *et al*., 2014). Importantly, it has been suggested that the expression of *GFAP* influences the ability of new neurons to integrate into existing neural circuitry, with *GFAP*‐knockout mice reporting higher rates of neurogenesis in the hippocampus (Larsson *et al*., 2004; Widestrand *et al*., 2007). In our study, we found a significant increase in *GFAP* expression with age, consistent with previous studies. As *GFAP* is expressed in both astrocytes and stem cells, we also quantified the expression of the mature astrocyte marker *S100B* and found it to be unchanged with age, suggesting that the observed change is associated with increased astrocyte activation rather than astrogliogenesis. This is supported by our observation that astrocyte morphology in our aged cases is consistent with that reported for activated astrocytes (Fig. 2h). Partial correlation analysis showed that neither *MKI67* (*r*(23) = −0.204; *P* = 0.327) nor *DCX* (*r*(23) = −0.342; *P* = 0.094) expression was associated with *GFAP* expression when controlling for age. This suggests that age‐related decreases in neurogenesis in the human hippocampus may not be a direct consequence of increased *GFAP* expression.

In this study, we provide data supporting the hypothesis that the stem cell population of the human hippocampus may be maintained throughout adult life. We also found that mRNA markers of proliferation and of nascent neurons are concomitantly, but potentially independently, reduced with age. Our data suggest that human hippocampal neurogenesis is altered at specific developmental stages in the aging human brain. We suggest that these stages may thus be appropriate points for the development of treatments which aim to restore neurogenesis and thus potentially support cognitive function.

## Author contributions

KM contributed to the conception and design of this study, experimental procedures, statistical analysis and writing of the manuscript. KA and DB contributed to the experimental procedures and editing of the manuscript. HB contributed to the statistical analysis and editing of the manuscript. CSW contributed to the conception and design of this study and editing of the manuscript. KD contributed to the conception and design of this study, statistical analysis and editing of the manuscript.

## Funding

This study was supported by the Discipline of Biomedical Science at the University of Sydney, the Schizophrenia Research Institute (utilizing infrastructure funding from the NSW Ministry of Health and the Macquarie Group Foundation), the University of New South Wales and Neuroscience Research Australia. CSW is a recipient of a National Health and Medical Research Council (Australia) Principal Research Fellowship (PRF) (#1117079). Tissues were received from the New South Wales Brain Tissue Resource Centre at the University of Sydney supported by the Schizophrenia Research Institute and the National Institute of Alcohol Abuse and Alcoholism (NIAAA).

## Conflict of interest

No conflicts of interest have been reported by any of the authors involved in the publication of this study.

## Supporting information


**Table S1.** Case demographics for qRT‐PCR and IF analysis.
**Table S2.** TaqMan probes used in qRT‐PCR analysis.

**Data S1.** Experimental procedures.
