# Supplementary material for: Evidence for reduced neurogenesis in the aging human hippocampus despite stable stem cell markers
Source: Aging Cell. 2017 Aug 1;16(5):1195–9. doi: 10.1111/acel.12641 (PMC5595679; doi:10.1111/acel.12641)
Supplement: Supplementary file 1 — Table S1. Case demographics for qRT‐PCR and IF analysis. Table S2. TaqMan probes used in qRT‐PCR analysis. [file ACEL-16-1195-s001.docx]

Supplementary Table 1 - Case demographics for both qRT-PCR and IF analysis

| Case Number | Age (years) | Gender | Cause of Death | PMI (hours) | pH | RIN | qRT-PCR | IF |
| --- | --- | --- | --- | --- | --- | --- | --- | --- |
| 1 | 18 | Male | Cardiac failure | 28.5 | 6.70 | 7.9 | x | x |
| 2 | 19 | Male | Trauma | 20.5 | 6.60 | 6.3 | x |  |
| 3 | 24 | Male | Cardiac failure | 43.0 | 6.27 | 6.7 | x |  |
| 4 | 29 | Female | Cardiac failure | 40.0 | 6.83 | 6.0 | x | x |
| 5 | 39 | Male | Cardiac failure | 22.0 | 6.49 | 7.2 | x |  |
| 6 | 47 | Male | Cardiac failure | 27.0 | 6.66 | 6.8 | x | x |
| 7 | 48 | Male | Cardiac failure | 17.0 | 6.71 | 7.8 | x |  |
| 8 | 49 | Male | Cardiac failure | 45.5 | 6.45 | 5.6 | x |  |
| 9 | 51 | Female | Cardiac failure | 37.5 | 6.92 | 6.2 | x |  |
| 10 | 52 | Male | Unknown | 36.0 | 6.82 | 8.0 | x |  |
| 11 | 53 | Male | Cardiac failure | 27.0 | 6.64 | 6.6 | x |  |
| 12 | 53 | Male | Cardiac failure | 26.0 | 6.36 | 5.5 | x |  |
| 13 | 54 | Male | Cardiac failure | 29.0 | 6.80 | 6.9 | x |  |
| 14 | 58 | Male | Cardiac failure | 39.0 | 6.49 | 6.8 | x |  |
| 15 | 59 | Male | Cardiac failure | 28.5 | 6.81 | 7.1 | x |  |
| 16 | 61 | Male | Cardiac failure | 22.0 | 6.41 | 6.4 | x |  |
| 17 | 62 | Male | Cardiac failure | 37.5 | 6.56 | 5.8 | x |  |
| 18 | 63 | Female | Cardiac failure | 50.0 | 6.46 | 6.5 | x |  |
| 19 | 64 | Male | Cardiac failure | 39.5 | 6.68 | 6.4 | x |  |
| 20 | 66 | Male | Cardiac failure | 32.0 | 6.66 | 8.0 | x |  |
| 21 | 73 | Male | Cardiac failure | 51.0 | 6.82 | 3.7ᵃ |  | x |
| 22 | 73 | Female | Cardiac failure | 45.0 | 6.86 | 6.9 | x |  |
| 23 | 74 | Male | Respiratory failure | 10.0 | 6.22 | 7.1 | x |  |
| 24 | 81 | Male | Cardiac failure | 29.0 | 6.57 | 6.2 | x |  |
| 25 | 83 | Male | Respiratory failure | 10.0 | 6.67 | 7.3 | x |  |
| 26 | 86 | Male | Infection | 15.0 | 6.94 | 6.9 | x |  |
| 27 | 88 | Male | Respiratory failure | 9.0 | 6.36 | 6.2 | x | x |
| Average | 56.6 | - | - | 30.2 | 6.62 | 6.73 | - | - |
| SEM (±) | 3.69 | - | - | 2.32 | 0.04 | 0.14 | - | - |
| PMI – post-mortem interval, RIN – RNA integrity number, qRT-PCR – Qualitative reverse transcriptase polymerase chain reaction, IF – Immunofluorescence,  SEM – Standard error of the mean, ᵃ - Case excluded in calculation of mean ± SEM due to RIN <5.0 | | | | | | | | |
|  |  |  |  |  |  |  |  |  |

Supplementary Table 2 - TaqMan probes used in qRT-PCR analysis

| Gene Name | Gene Symbol | NCBI Reference Sequence | TaqMan Assay ID |
| --- | --- | --- | --- |
| Glial fibrillary acidic protein, isoform delta | *GFAPδ* | NM_001131019.2 | AILJJYD |
| Ki67 | *MKI67* | NM_002417.4 | Hs01032443_m1 |
| Eomesodermin | *EOMES* | NM_001278182.1 | Hs00172872_m1 |
| Doublecortin | *DCX* | NM_000555.3 | Hs00167057_m1 |
| Glial fibrillary acidic protein, isoform alpha | *GFAP* | NM_002055.4 | Hs00909233_m1 |
| S100 calcium binding protein B | *S100B* | NM_006272.2 | Hs00902901_m1 |
| Importin 8 (housekeeping gene) | *IPO8* | NM_001190995.1 | Hs00183533_m1 |
| TATA-box binding protein (housekeeping gene) | *TBP* | NM_003194.4 | Hs99999910_m1 |
| Ubiquitin C (housekeeping gene) | *UBC* | NM_021009.5 | Hs00824723_m1 |
