# Supplementary material for: Evidence for reduced neurogenesis in the aging human hippocampus despite stable stem cell markers
Source: Aging Cell. 2017 Aug 1;16(5):1195–9. doi: 10.1111/acel.12641 (PMC5595679; doi:10.1111/acel.12641)
Supplement: Supplementary file 2 — Data S1. Experimental procedures. [file ACEL-16-1195-s002.docx]

**Experimental Procedures**

*Human post-mortem brain tissue*

Post-mortem tissue form the anterior hippocampus was provided from 29 healthy individuals aged between 18 and 104 years (New South Wales Tissue Resource Centre, Sydney, Australia). Each case was free of significant neuropathology upon post-mortem examination. Fresh frozen tissue was collected from all 29, of which 26 were used in the final analysis. Additionally, formalin fixed paraffin embedded (FFPE) hippocampus tissue was also obtained from a subset of cases of the full cohort aged 18-88 (n=5), taken from the opposite brain hemisphere to the frozen tissue (Supplementary Table 1). This study was approved by the Human Research and Ethics Committee at the University of Sydney (USyd HREC 2015/584).

*RNA extraction and cDNA synthesis*

Fresh frozen tissue was supplied in 60 µm thick sections, from which the hippocampus was dissected and collected for RNA extraction. Briefly, 800 µL of TRIzol reagent (Thermo Fisher Scientific; 15596018) was added to 50-100 mg of tissue. The tissue and reagent were homogenised manually with a disposable pestle and incubated at room temperature for 5 minutes to allow dissociation of the nucleoprotein complex. 160 µL of chloroform was added to each sample and vortexed briefly to mix. After incubating for 3 minutes at room temperature, the samples were spun in a centrifuge at 12,000 xg (15 mins, 4°C) to facilitate phase separation. The aqueous phase was pipetted into a new tube and the RNA precipitated from solution by adding 500 µL of isopropanol and incubating for 10 minutes at room temperature. The RNA/isopropanol mixture was centrifuged a second time at 12,000 xg (10 mins, 4°C). The supernatant was poured off and the resulting RNA pellet resuspended in 500 µL of 75% ethanol. The tubes were then centrifuged a third time at 7500 xg (5 mins, 4°C). The supernatant was poured off and the RNA pellet allowed to air dry. The dry pellet was then resuspended in 50 µL of RNase-free water and stored at -80°C until further use. RNA concentration and purity was determined using the NanoDrop ND-1000 spectrophotometer (Thermo Fisher Scientific, Massachusetts, USA) and RNA integrity number (RIN) by the Agilent 2100 Bioanalyser (Agilent Technologies, California, USA). Samples with a RIN less than 5.0 (n=3) were excluded from mRNA analysis due to poor RNA quality, the average RIN of the remaining cases used for all statistical analyses was 6.73 ± 0.14.

cDNA was synthesised using the SuperScript III First-Strand Synthesis SuperMix kit (Invitrogen; 18080400). 2 µg of RNA from each case was combined with 1 µL of both random hexamers and annealing buffer. RNase-free water was added to bring the final volume to 8 µL. Samples were incubated in a thermocycler at 65°C for 5 minutes before being placed immediately on ice for at least 1 minute. 10 µL of 2x First-Strand Reaction Mix was added, followed by 2 µL of ice-cold SuperScript III/RNaseOUT Enzyme Mix. Samples were then immediately incubated in a thermocycler for 5 mins at 25°C followed by 50 mins at 50°C. Synthesised cDNA was kept at -20°C for short-term and -80°C for long-term storage. A second round of cDNA synthesis was also completed using eight randomly-selected cases from the entire cohort. This cDNA was pooled and serially diluted (3x) to create standards against which the final mRNA concentration of each case was measured.

*Quantitative reverse transcriptase polymerase chain reaction (qRT-PCR)*

mRNA expression of neurogenesis and gliogenesis markers were measured using TaqMan Gene Expression Assays (Applied Biosystems; Supplementary Table 2). TaqMan probes were selected to measure the expression of the genes *GFAPδ* (glial fibrillary acidic protein isoform δ; stem cells), *MKI67* (Ki67; cell proliferation), *EOMES* (eomesodermin; neuronal progenitors), *DCX* (doublecortin; immature neurons), *GFAP* (glial fibrillary acidic protein isoform α; astrocytes and stem cells) and *S100B* (S100 calcium-binding protein B; mature astrocytes). Data were collected using an ABI Prism 7900HT fast real-time PCR system in a 384-well format and captured using sequence detector software (SDS version 2.4; Applied Biosystems). All measurements were made in triplicate, and relative quantities determined using a seven-point standard curve using the pooled and serially diluted cDNA described above. The expression of three housekeeping genes was also measured; importin 8 (*IPO8*), TATA binding protein (*TBP*) and ubiquitin C (*UBC*), with mRNA expression of the target genes normalised to the geomean of the housekeeping genes. This geomean was not significantly correlated with age in the hippocampus (r=-0.187; p=0.361).

*Immunofluorescence*

Immunofluorescence (IF) staining was performed on 10 µm thick FFPE sections to examine changes to the expression of GFAP in astrocytes. Briefly, slides were deparaffinised in xylene and taken through graded alcohol to water. Antigen retrieval was performed by incubating slides in 10mM citrate buffer (pH 6.0) at 95°C for 30 minutes, followed by 30 minutes cooling at room temperature. Slides were washed 3x in 50% ethanol before peroxidase quenching in 0.3% H₂O₂ in phosphate buffered saline (PBS; pH 7.4) for 30 mins. Slides were then blocked for 1 hour at room temperature in blocking buffer (0.5% casein, 1% bovine serum albumin, 0.05% Tween20 in PBS). Primary antibody (Ms α-Hu GFAP, 1:7000; Sigma, G3893) diluted in blocking buffer was applied and the slides incubated at 4°C overnight. Sections were washed 3x in PBST (PBS plus 0.1% Tween 20) before incubation with horseradish peroxidase-conjugated secondary antibody (α-Ms HRP, 1:1000; Chemicon, AP308P) for 2 hours at room temperature. After washing, a tyramide signal amplification kit was used, and the slides incubated with Cyanine 3 according to the manufacturer’s instructions (PerkinElmer, NEL752001KT). Nuclear counterstaining with 4’, 6-diamnidino-2-phenylindole (DAPI) was performed (1:750; 10 minutes) before coverslipping with 80% glycerol in PBS. Coverslipped slides were stored in the dark at 4°C.

Images of the stained sections were taken using a Zeiss LSM 710 confocal microscope, Zeiss Axiocam HRm camera and Zeiss ZEN v11.0 capture software (Zeiss, Oberkochen, Germany). Three randomly selected areas of the hippocampal hilus were photographed at 200x magnification for visual analysis.

*Statistical Analysis*

All statistical tests were performed using IBM SPSS Statistics v22. Normalised mRNA expression was analysed using Spearman’s rank correlation coefficient, with a confidence interval of 95%. The potential correlative relationship between two experimental variables, while controlling for a third experimental variable included as a covariate, were examined using partial correlation. This examined the effects of *MKI67* and *DCX* controlling for age and *GFAPδ* and age controlling for total *GFAP* expression. Graphs were created in GraphPad Prism 6 (GraphPad Software, California, USA).
